# Ecological legacy of Indigenous dispossession: disruption of ancestral sea gardens by commercial clam fisheries in British Columbia, 1882–1985

**DOI:** 10.1098/rstb.2024.0277

**Published:** 2025-07-10

**Authors:** Patrick Hayes, Skye Augustine, Marco Hatch, Loren McClenachan

**Affiliations:** ^1^ School of Environmental Studies, University of Victoria, Victoria, British Columbia V8P 5C2, Canada; ^2^ Department of History, University of Victoria, Victoria, British Columbia V8P 5C2, Canada; ^3^ Trinity Centre for Environmental Humanities, Trinity College Dublin, Dublin D02 PN40, Ireland; ^4^ School of Resource and Environmental Management, Simon Fraser University, Burnaby, British Columbia, Canada V5A 1S6; ^5^ Centre for Indigenous Fisheries, The University of British Columbia, Vancouver, British Columbia, Canada V6T 1Z4; ^6^ Western Washington University, Bellingham, WA 98225, USA

**Keywords:** clam gardens, Indigenous fisheries, marine historical ecology, environmental history, fisheries management, traditional knowledge

## Abstract

Ancestral sea gardens in British Columbia (BC) were managed to provide high long-term yields for millennia before European contact. While the ubiquity and productivity of sea gardens are well known, the ecological and cultural impact of the transition from ancestral to settler-colonial fisheries has not been investigated in a historical ecology context. Here, we examine that transition in the BC clam fishery, documenting the social and legal mechanisms used to dispossess Indigenous clam harvesters and identifying the ecological impacts of settler-colonial fisheries. Our results document a rapid change in clam tending, harvesting and processing that relied on Indigenous land, knowledge, labour and skill being directed away from traditional harvesting and towards unsustainable catches for commercial gain. We identify evidence of serial depletions and declines in relative abundance as measured by spatial shifts in harvesting, declines in catch per unit of effort, and a transition from native to non-native clam species. Finally, we identify additional evidence of the legacy of Indigenous tending 50 years after the start of the commercial fishery, demonstrating sustained productivity on beaches that continued to undergo Indigenous harvesting. Ongoing efforts in BC aim to restore ancestral clam beaches and tending practices. Historical insights can play a crucial role in restoration efforts by identifying ecological baselines and helping support the Indigenous reclamation of ocean spaces and the responsibility to tend them.

This article is part of the theme issue ‘Shifting seas: understanding deep-time human impacts on marine ecosystems’.

## 1. Introduction

Historical marine ecology has documented extensive declines in marine ecosystems globally [1–3]. In regions with colonial histories, these declines are often linked to transitions from Indigenous to settler-colonial systems of exploitation and resource management [4–6]. One well known case is that of Indigenous sea gardens in the Pacific northwest (PNW), which were managed to provide high yields over generations for thousands of years before European contact. While the ubiquity and productivity of sea gardens and ancestral clam tending in the PNW have been well documented, the transition to settler-colonial fisheries and the ecological and cultural impact of that transition has not been investigated in a historical ecology context.

Sea gardens (also known as clam gardens) are sites where Indigenous Peoples from what is now called Washington State, coastal British Columbia (BC), and southeast Alaska have modified the intertidal environment to enhance food system resilience for at least the last 3500 years [7–10]. Sea gardens are commonly constructed by building a rock-walled terrace at the low tide line, flattening the beach and increasing clam habitat. The modified beach increases clam growth rates, density and harvestable biomass [11]. The rock terrace increases three-dimensional structure, providing habitat for additional traditional food species (e.g. sea cucumbers, chitons, whelks) [12]. The unique sediment structure moderates thermal stress, potentially leading to higher survivorship from heatwave events [13,14]. These practices, combined with other forms of active cultivation such as predator removal, and designated access rights, ensured stable and abundant clam populations over millennia [15].

Sea gardens are also cultural keystone places where communities connect with the intertidal [16]. They are places where Indigenous rights and title are expressed, where elders take youth to share knowledge of reciprocal relationships with the natural world, and where responsibilities to care for beaches are practised and passed down. However, the active management and tending of sea gardens has been interrupted in many communities resulting from the many atrocities faced by Indigenous Peoples of the PNW, including waves of disease, violent removal from the land, banning of cultural practices and forced attendance of boarding schools [17].

Ongoing efforts in the PNW aim to restore ancestral clam beaches and tending practices. These restoration efforts are grounded in Indigenous Knowledge and focused on traditional harvesting spots that once held high densities of native bivalve species. However, in many cases, these efforts now exist in the context of colonial impacts that led to the decline of beaches. The impacts since European contact in this region are myriad and, in some cases, can be challenging to define. The dispossession of these beaches from Indigenous Peoples by settler-colonial institutions and agencies is the most widely referenced cause for the observed declines in sea garden productivity owing to the sudden widespread absence of active stewardship practices (which encompasses First Nations' roles, responsibilities and care of beaches) that had been applied *in situ* by Indigenous Peoples for millennia [7,9,11,18,19]. There may also be site-specific impacts from modern industrialization and urban development, such as log booming, ferry wakes, contaminated effluent/runoff and shoreline development [18], and more regional/global phenomena that are causing difficult-to-quantify impacts on native species and shoreline ecosystems, such as climate change and ocean acidification [20]. While these various impacts and stressors likely compound the continued declined state of some native bivalve species following the disruption in Indigenous stewardship, the scale, magnitude and interrelationship among contributing factors have yet to be fully defined and documented. Building a more detailed picture of the impacts that precipitated rapid declines in a species can lend valuable insight to inform strategies for species and ecosystem recovery.

Other studies have noted dramatic declines in clam abundance following European contact in 1782 [15], yet historical documentary evidence related to the decline of Indigenous clam tending remains underexamined. Here, we use historical documentary records to investigate the transition from Indigenous to settler-colonial clam fisheries from the late nineteenth century onwards. We aim to document the ecological impacts of settler-colonial fisheries and the social and legal mechanisms that dispossessed Indigenous clam harvesters and disrupted millennia-old practices that promoted increased clam productivity. Our goal is to aid restoration efforts by identifying ecological baselines and synthesizing historical evidence of commercial industry impacts, reinforcing the need for Indigenous reclamation of ocean spaces.

## 2. Background

**Figure 1.**
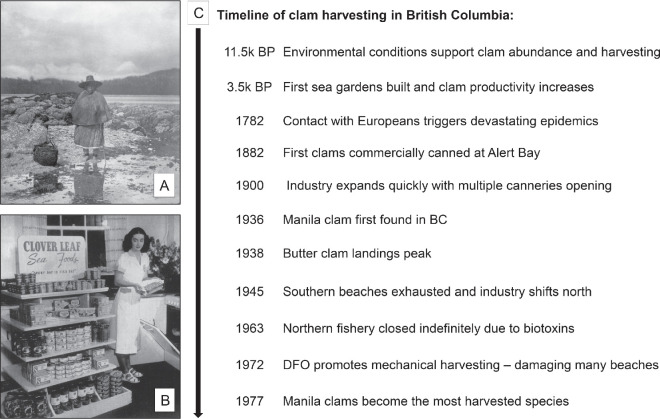
Historical photographs and timeline of clam harvesting in BC: (A) a high-born Kwakwaka'wakw clam-digger, 1915 [21], (B) display of ‘Clover Leaf’ products (clams bottom shelf) [22], (C) key events in BC clam harvesting. BP, Before Present.

Indigenous Knowledge, archaeological and ethnographic research indicate that clams, particularly butter clams (*Saxidomus gigantea*) and littleneck clams (*Protothaca staminea*), were an abundant and essential food source for northwest coast Indigenous Peoples for millennia before European contact (see figure 1 for timeline of clam harvesting in BC) [18]. Favourable conditions for butter clams emerged in the PNW around 11 000 years ago, with human–clam relationships intensifying at least 3500 years ago, when some of the first clam gardens were constructed [10]. However, the number of sites that have been carbon-dated remains small, and the practice of clam garden construction and beach cultivation could extend much further back in time. Archaeological findings from Quadra Island, BC, show that harvesting pressure on clams increased during this period, yet there is no evidence of declining abundance or serial depletion [15]. This was largely owing to the construction of clam gardens, which expanded viable habitat and sustained productivity. Indigenous communities also employed a range of other techniques to enhance clam abundance and size, many of which are discussed in more detail in §4a [15].

From the late eighteenth century onward, Indigenous Peoples in BC experienced devastating impacts owing to European-introduced diseases, colonial policies aimed at cultural erasure and community destruction, and the dispossession of land and sea rights [15]. Introduced by settlers, epidemics such as smallpox (first appearing soon after contact in 1772 and returning in 1862−1863), measles (1847−1850) and tuberculosis decimated Indigenous communities and caused dramatic population decline [23–26]. Disease impacts were compounded by banning cultural practices, such as the Potlatch ban in 1844, splitting ancestral land by the USA/Canada border in 1846, and forced attendance of residential schools from 1920 onwards [26,27]. These atrocities fundamentally altered traditional clam tending practices, as the social and ecological systems that supported sustainable management were severely affected [15]. The beginning of a settler-controlled commercial clam fishery in 1882 further disrupted these systems.

**Figure 2.**
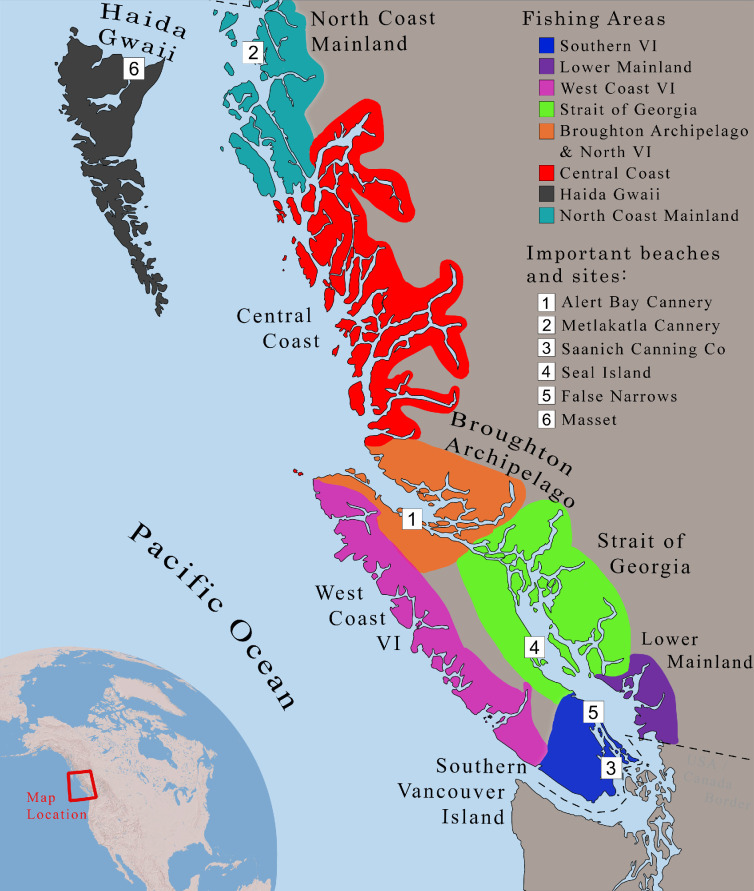
Study area and key sites. Colours correspond to areas in figure 3. VI, Vancouver Island.

Despite these challenges, Indigenous communities continued to rely heavily on traditional clam harvesting and adapted to the commercial industry. Most remained resilient, continuing to tend ancestral beaches to varying degrees, but faced continuous disruption and threat from colonial institutions and settlers. For example, the K'ómoks First Nation, whose traditional territory is in the Strait of Georgia region, continued to tend their ancestral beaches, yet some of their most important sites were closed by the Department of Fisheries in Canada, known as Fisheries and Oceans Canada (DFO). Likewise, the Haida people played a central role in the commercial razor clam (*Siliqua patula*) fishery on Masset Beach at the northern tip of Haida Gwaii (figure 2, location 6). The Kwakwaka’wakw, whose traditional territory is in the Broughton Archipelago, adapted to the commercial fishery and continued to assert control over clam harvesting [19].

**Figure 3.**
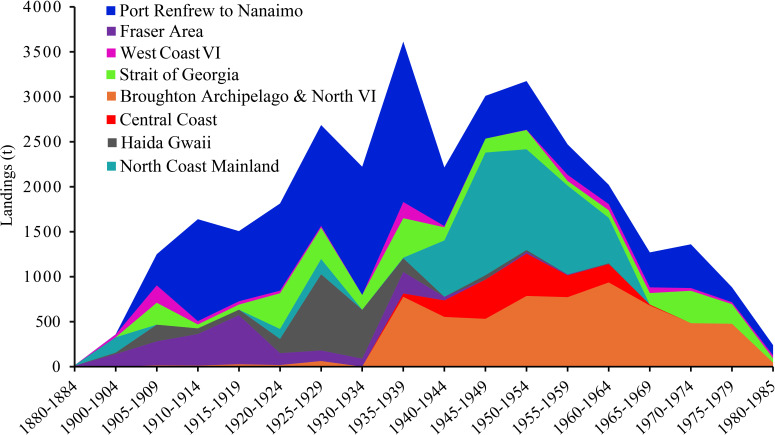
Butter and littleneck clam landings by area, 1880−1985. Landings have been grouped over 5-years periods. Regions ordered south to north—Southern Vancouver Island (VI) most southerly and North Coast Mainland most northerly regions. Data from *Annual Reports of the Department of Marine and Fisheries, 1882−1946*, *Fisheries Statistics of Canada, 1918−1929*, *Provincial Department of Fisheries Reports, 1942−1947*, DFO, *Intertidal Clam Resources Report* vols I–III. See “Clam Landings & CPUE Data” in the Supplementary material for full references [28].

Canneries were major drivers of commercial exploitation. The first clam cannery to process clams opened in the Broughton Archipelago (Alert Bay, 1882−1884) in 1882, followed by another on the North Coast Mainland in 1883 (Metlakatla, 1883−1887). Other important canneries later processed clams in Southern Vancouver Island (VI) (i.e. Saanich, 1905−1942). While Indigenous people were involved in the operation of these canneries, they were all settler-owned. British Columbia’s proximity to the United States also influenced clam exploitation, with dynamics related to cross-border trade and serial depletion. For example, most clams canned in BC were exported to the United States, so a 1929 import tariff on canned clams impacted the Canadian industry [29]. By the mid-1930s, severe depletion of clam beds in Washington drove American harvesters to seek clams in BC [29,30]. Fuelled by strong demand from BC and US canneries, the clam fishery peaked in southern BC in 1938. Clam populations collapsed in southern BC soon after this peak, and the fishery shifted northwards.

In addition to the many other ecological and social disruptions outlined in this study, the increasing prevalence of harmful algal blooms (HABs) from the 1940s onwards severely disrupted clam harvesting, rendering many clam populations unsafe for consumption and contributing to long-term declines in access and availability. HABs can lead to the accumulation of biotoxins in shellfish, potentially causing paralytic shellfish poisoning (PSP) and other illnesses in individuals who consume contaminated seafood. PSP is caused by saxitoxin, a potent neurotoxin created by the naturally occurring dinoflagellate *Alexandrium* spp.*,* which can lead to severe health complications and even death [31]. HABs have always occurred in the PNW, but have likely increased in frequency and severity since the arrival of settlers [32–34]. The first settler record of PSP illness in the PNW is from members of Captain George Vancouver’s Royal Navy crew who became ill in 1793 after consuming shellfish harvested from Poison Cove in southeast Alaska, USA [35]. There are few other documented cases of illness or death from shellfish poisoning before 1940; however, after this date, health authorities in BC and Washington State began to report sporadic cases (see §4b(i) for more). The impacts of HABs and bacterial/viral pathogens continue to create accessibility challenges owing to shellfish harvesting closures that disproportionately affect Indigenous communities through eco-colonization [36,37].

One of the most dramatic area closures occurred in 1963, when all harvesting north of the Broughton Archipelago was prohibited owing to high levels of marine biotoxins [38]. Butter clam landings in BC were minimal after 1963. The Broughton Archipelago and Strait of Georgia regions continued to produce harvests into the 1970s, but at far reduced levels. Around this time, DFO also promoted the use of mechanical harvesting, which likely irrevocably damaged many beaches and reduced landings further [38]. Clam landings in BC revived in the 1980s, but this fishery was very different from what came before. The new fishery was focused on the Manila clam (*Venerupis philippinarum*), which was accidentally introduced to BC in the 1930s [39]. This new fishery was primarily based on commercial aquaculture, with most landings coming from the Strait of Georgia [40]. The Manila clam retains toxins for far less time than butter clams, meaning it is safer to consume [34]. By the time the Manila clam became dominant in the 1980s, commercial fisheries for native species had all but ceased.

## 3. Methods

### (a) Positionality statement

A multidisciplinary team conducted this study, each bringing unique perspectives to the research. Lead author Patrick Hayes is an Irish historian temporarily based in Canada who specializes in marine environmental history. He led the historical documentary research, focusing on archival and colonial sources to uncover the impacts of the settler-colonial clam fishery. Second author Hwsyun’yun Skye Augustine, is from the Stz’uminus Nation and is an Assistant Professor in the Centre for Indigenous Fisheries at the University of British Columbia. Skye is a leader in reawakening sea garden practices, and Indigenous governance and stewardship. Third author Marco Hatch is a member of the Samish Indian Nation and an American marine biologist and professor known for integrating traditional ecological knowledge with contemporary marine science. Senior author Loren McClenachan is a historical marine ecologist of settler ancestry whose contributions included the identification of methodologies for integrating qualitative and quantitative sources and analysing historical information in an ecological context.

### (b) Study area

This study focuses on the coastal region of British Columbia, a province in western Canada. We split the coast into eight separate areas based on historical and modern DFO fishery management areas (figure 2). BC is home to a great diversity of Indigenous Peoples, with numerous distinct languages, traditions and governance systems.

### (c) Colonial documentary sources

Our study examines the colonial historical records to better understand the transition from Indigenous to settler-colonial clam fisheries. We recognize the inherent biases and limitations of colonial archival sources. These records, predominantly created and maintained by settlers, often reflect the perspectives and interests of colonial authorities while marginalizing and misrepresenting the experiences and knowledge of Indigenous Peoples. We acknowledge that much of what we discuss here as historical data represents Indigenous communities' lived experiences and ongoing realities. We approach this research with a commitment to respect Indigenous voices and knowledge systems and acknowledge the need to critically analyse and contextualize colonial records.

We systematically searched published primary sources and archives for records related to social and ecological history of clam harvesting in local BC archives. We also extensively used DFO archival records from BC Archives and Department of Indian Affairs and DFO records from Library and Archives Canada, as well as printed primary sources from DFO and other institutions. We also reviewed publicly available records that directly quote Indigenous Knowledge Holders or were written by Indigenous authors. Though many of the impacts described in this study are well documented in the public record, this work refers to available records from Indigenous Knowledge Holders, harvesters and beach stewards.

We also conducted a consultation process with many First Nations connected to the historical records used in this study. Through these engagements, we gathered input and feedback that informed and enriched our research. This process also led to the acquisition of additional information, including from the K’ómoks First Nation, with whom we established a research agreement, and shared findings related to the historical destruction of cultural shell deposits (see §4a(iv) for more). Our process has far from captured the full spectrum of Indigenous Knowledge related to clam harvesting. Future work could look to conduct interviews or work more closely with Indigenous Knowledge Holders to elucidate more detail and nuance to the patterns seen in the historical records we examined.

To examine the social and legal mechanisms used to dispossess Indigenous clam harvesters, we analysed documents related to clam management, harvesting, processing and shell use. To document the ecological impacts of settler-colonial fisheries, we reconstructed historical clam landings from the beginning of the commercial fishery until the year 2022 and analysed these spatially and by species to look for evidence of serial depletion. To provide a measure of the relative abundance of clams, we also gathered historical catch per unit effort data from DFO sources. Finally, we identified evidence of continued ancestral tending practices. Further details on the sources and methods used can be found in the supplementary material.

We provide a supporting document with some additional information and a spreadsheet of our landings and catch per unit effort data for this study. Additionally, to support future research and the accessibility of records, we have compiled a metadata list of historical records found for this study. All these files are available on Figshare .

## 4. Results

### (a) Social change

**Figure 4.**
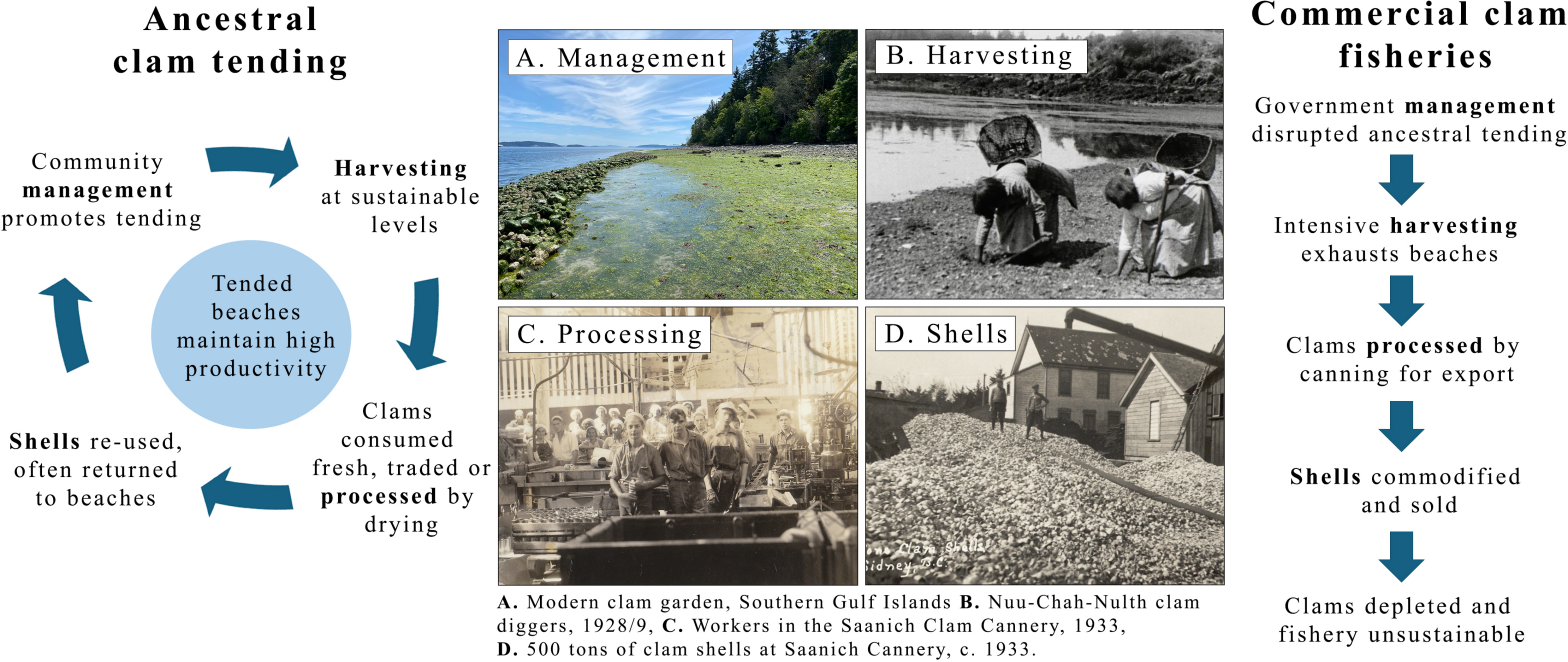
Ancestral clam tending compared with commercial clam fisheries across four components of clam production: (A) management [41], (B) harvesting 42], (C) processing [43] and (D) shell use [44].

Our results document rapid changes in clam management, harvesting, processing and shell use. We found that the settler-colonial clam fishery relied on Indigenous land, knowledge, labour and skill being directed away from ancestral clam tending and towards unsustainable catches for commercial gain (figure 4).

#### (i) Clam management

Beach management practices vary among First Nations and cannot be fully captured in a single definition, but they commonly emphasize familial and community responsibility for tending specific locations to maintain long-term productivity. For example, in W̱SÁNEĆ Nations (located in southern BC), many clam beaches are held by family units, while other sites are shared between families. W̱SÁNEĆ Elders and Knowledge Holders recount that ‘Belonging to a harvesting site creates a responsibility to the family for the care of the site’ [45]. Another Coast Salish group, the Samish, managed horse clam harvest at the family level, with family tenure of specific harvest areas within a larger beach area [46]. Through this proprietorship, often inherited along familial lines, there was an incentive to cultivate productive and healthy beaches for the benefit of present and future generations [46,47].

**Figure 5.**
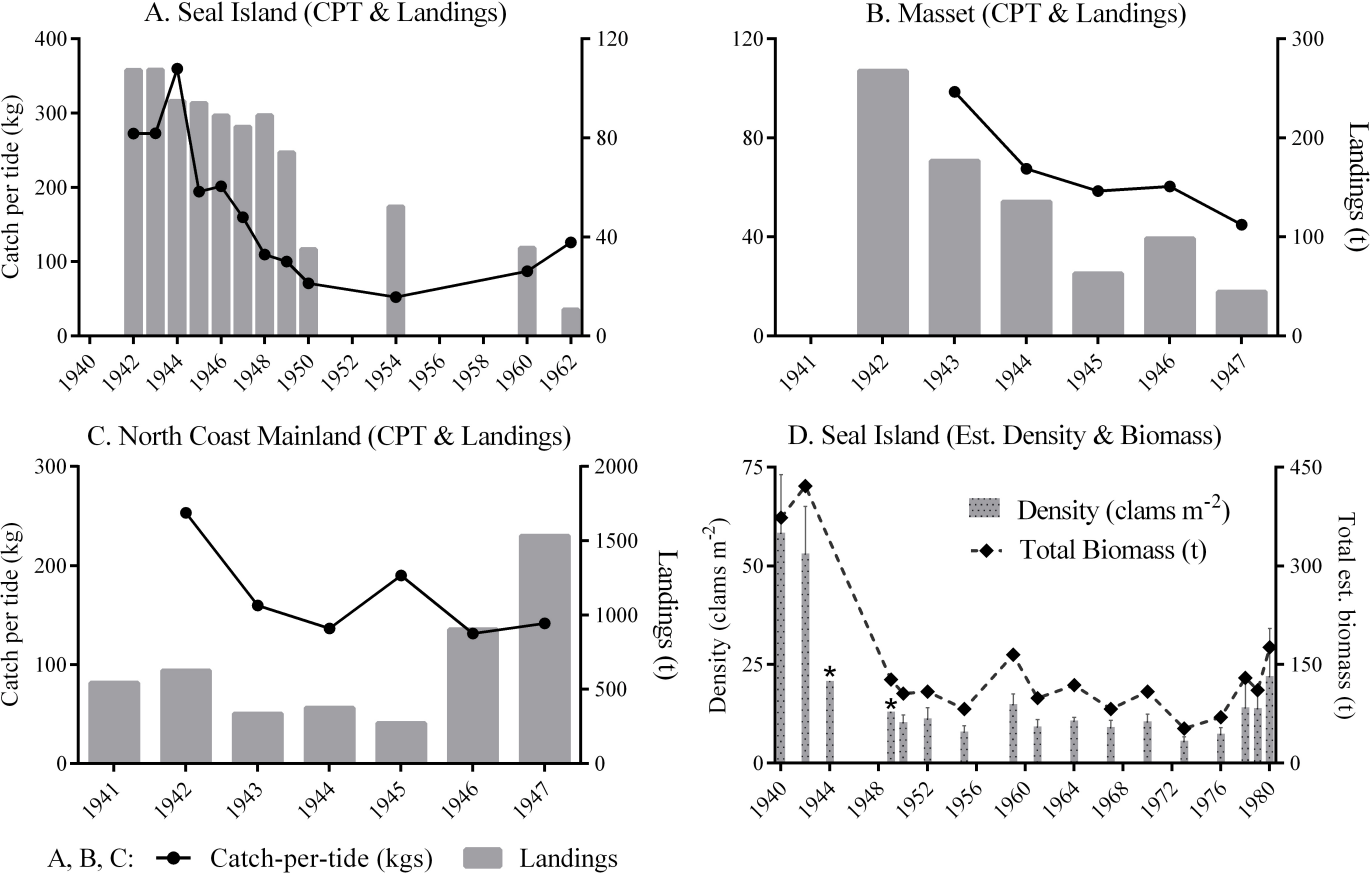
(A–C) Average catch per tide (CPT) and landings, 1940−1962. (D) Estimate of the population of legal-sized (>63 mm) butter clams at Seal Island, 1940−1962. Bars represent clams per square metre (left axis). Error bars indicate 95% confidence intervals; *error estimate not available. Dashed line represents the mean estimate of biomass of legal-sized clams in tonnes. Data from [51–53].

The colonial legal system disrupted ancestral clam tending by making most beaches open access and placing others under exclusive use for settlers only. Under English common law, the Crown assumed the right to the foreshore and guaranteed a public right to fish, meaning anyone could harvest clam beaches and benefit from the productivity generated through Indigenous tending, incentivizing rapid harvest. Additionally, the BC government retained the right to lease beaches to groups and individuals for exclusive harvesting, and DFO could close beaches and enforce harvesting bans. The first regulations created by the colonial government in 1910 were directed at trade regulations rather than conservation, with the banning of the export of raw clams to the USA [48]. In 1918, a closed season was implemented for June and July, and in 1922, minimum size limits were introduced [49,50]. These harvest regulations were regularly amended in later decades but failed to prevent the serial depletion of clam populations (see figures 2 and 5).

Colonial laws systematically prevented Indigenous communities from obtaining exclusive rights over ancestral beaches. From at least 1905, the federal Department of Justice ruled that Indigenous communities had no exclusive rights over beaches fronting their reserve land [54]. The Department of Indian Affairs tried to prevent leasing such beaches, but they were not always successful [55]. For example, in 1940, a settler fishing company was approved for a lease fronting the Kitasoo Indian Reserve [54]. The Department of Lands, responsible for approving leases, actively opposed granting Indigenous communities greater control. In 1930, the Department expressed its intent to reserve foreshore areas not only for settler-controlled clam beds but also because these areas could be ‘highly desirable in the future for industrial or other uses’ [19]. Even when ancestral beaches were not leased, they remained open access and were frequently overharvested by settlers [56]. Some Indigenous communities attempted to use trespassing laws to prevent outside harvesting, but in every case we examined, rulings went against these communities [57]. There are also many cases of Indigenous harvesters being forcibly removed from beaches by settlers at gunpoint, as well as incidents where DFO officers confronted harvesters and deliberately dumped their harvested clams [19].

Before 1960, Indigenous communities that attempted to obtain leases to ancestral clam beaches were denied tenure in all the cases we found. Instead, leases were given to settlers, or DFO closed the area for harvesting [58,59]. The case of Seal Island (figure 2, location 4) is indicative of this pattern. Seal Island is an ancestral beach of the K'ómoks First Nation, but from the late nineteenth century, the BC government claimed ownership [60]. Repeated lease requests by members of the K'ómoks First Nation in the 1930s were never granted [61]. In 1938, DFO closed Seal Island to harvesting, citing conservation concerns [62,63]. In 1943, the K'ómoks Nation tried again, requesting an ‘undisputed right’ to harvest clams on the beach [64]. DFO denied the request [65], with one DFO official claiming the K'ómoks were only interested in the beach ‘thanks to the efforts of the Fisheries Department’ in restoring the clam population [66]. DFO claimed the local K'ómoks community could continue harvesting clams from the beach for food while it was closed, but they also reported prosecuting two locals for ‘digging clams commercially’ [65]. Other evidence shows that clam abundance declined on the beach following its closure by DFO (see §4b(ii)).

#### (ii) Clam harvesting

Traditionally, Indigenous communities employed harvesting practices to achieve long-term yields over multiple generations; the commercial fishery incentivised boom-and-bust harvesting, exhausting beaches before seeking new clam sources. Kwakwaka’wakw Clan Chief Kwaxsistalla Wathl’thla, Adam Dick, explains that the Kwakwaka’wakw used yew-wood sticks to loosen beach sediments and pry up clams, making it easier to use traditional tools and aerating the substrate to encourage clam growth. Ancestral harvesters were carefully trained to harvest only mature clams of a certain size and dig slowly in a wide area to prevent localized overharvesting [67,68]. Evidence from cultural shell deposits on Quadra Island showed that 63% of harvested clams were above the modern size limit of 63 mm [15]. Similar harvesting practices were employed by other First Nations and were vital in helping clam gardens become more productive over time [13,69].

Indigenous clam diggers who worked in the commercial clam fishery continued to use traditional methods. A 1912 report on the status of clams in British Columbia noted that Indigenous clam diggers in Burrard Inlet, just north of Vancouver, continued to dig by hand but had replaced wooden sticks with steel blades. The same report noted that settlers and some Indigenous harvesters used spades or potato forks for digging in other areas [70]. These methods persisted throughout the history of the commercial clam industry [38].

There were repeated attempts from settler-controlled canneries to introduce mechanical clam diggers in order to increase harvesting efficiency, lower labour costs, and in some cases, increase output from beaches with falling productivity. For example, in 1930, the Langara Packing Company tested a machine they claimed would increase their output by ‘100% without causing any depletion’ [71]. However, they faced opposition from local Haida harvesters, who stood to lose their livelihoods owing to mechanization and likely had concerns about its long-term sustainability. Ultimately, the machine never functioned efficiently enough to be used commercially [72]. This proposal, and many others [73], met strong opposition from DFO, which had concerns about mechanical harvesters’ ability to filter out immature clams [74,75]. A DFO scientist from the time also noted that mechanical diggers would take the fishery ‘out of the hands’ of Indigenous communities who did most of the harvesting [76].

In the 1950s, the fishing regulations banned mechanical diggers in BC, but the ban was lifted in 1964 when a permit system was introduced [38,77]. By the 1970s, DFO had changed stance and was promoting the use of mechanical harvesting as a means to increase dwindling clam landings. In 1972, DFO scientists D. B. Quayle and N. Bourne called for the greater use of mechanical diggers, claiming they were needed owing to a shortage of skilled harvesters. They wrote, ‘if commercial clam landings are to increase, the digging methods must be greatly improved by mechanization’. They advocated the use of hydraulic diggers that employed pressurized water jets to liquidize beach sediment, which would allow clams to float to the surface. They noted that single fire hoses were only recommended on rockier beaches, as otherwise they could create deep gouges in the beach [38, p. 63].

Many Indigenous communities have reported that beaches were irrevocably damaged by mechanical digging, particularly by fire hoses [78]. Many other proposals for mechanical harvesters were made in the 1970s and 1980s, many of which were opposed by local communities [19]. By 1986, DFO abandoned its efforts to implement mechanical harvesting, which was subsequently banned outright [78].

Commercial harvesters prioritized rapidly extracting clams from a beach until it was no longer economically profitable, after which they moved on to the next area. This pattern was largely driven by the demand for clams from settler-owned canneries. A DFO report published in 1913 recounts that a cannery had previously operated from a barge, completely depleting one clam bed before moving on to the next. DFO scientists described this harvesting method as ‘dangerous’, warning that it led to each cannery ‘taking all that can be obtained in any one place, with the promise of other beds to be had for the trouble of moving’ [70, p. 44]. To incentivize sustainable harvest and create a ‘spirit of conservation’, DFO scientists in the 1910s and 1930s suggested canneries be given leases with ‘the retention of this area be made dependent on the abundance of clams in the beds’ [70,79]. Although never implemented, these ideas mirror the place-based governance structures that helped manage clam gardens for millennia, but with the goal of ensuring a steady supply of clams to settler-owned canneries. The same scientists opposed leasing clam beds to Indigenous communities, arguing these communities had no ‘understanding as to why the yield of a [clam] bed should be regulated’ [80, p. 112].

#### (iii) Clam processing

Clams were traditionally harvested and preserved for local use or trade, guided by principles of reciprocity and multigenerational resilience. This system was fundamentally disrupted by the commercial clam fishery, which redirected harvests to settler-controlled canneries supplying expansive domestic and export markets. These canneries, driven by industrial demands, incentivized large-scale exploitation with little regard for local depletion, relying instead on the mobility of the industry to continually move operations to new, unexploited areas. In contrast, ancestral harvesting and processing practices were place-based and focused on sustaining the long-term needs of the communities who tended the beaches.

The clam canning industry changed how clams were processed and consumed by creating a long-lasting product that could be marketed and sold to customers worldwide (see figure 1B for an example of canned clam advertising). Approximately 57% of all butter clams and littleneck clams harvested in BC before 1946 (the end of available statistics) were canned in the province. The remaining 43% were sold fresh locally or exported to the USA for canning there.

Some Indigenous communities attempted to process clams within the economic and legal system imposed on them. For example, the cannery at Metlakatla was run cooperatively by Indigenous people, although settlers held all the management positions [81]. In 1914, Chief Tsukaite of the Nahkwockto Band asked a visiting representative of the McKenna-McBride Commission for help in establishing a cannery for fish and clams, explaining that since settlers had cut all the timber there were few economic opportunities, and the cannery would provide ‘something to fall back on when they came to be squeezed out of all the work at the white men’s canneries’ [82, p. 136]. This proposed cannery was never opened. In the mid-1940s, the Masset Co-Operative Association took over harvesting at Masset beach (figure 2, location 6), with local Haida people buying shares in the company; the business lasted until the 1960s [83–85]. These cases are exceptions, and almost all clam canneries in BC were owned by settlers. For example, the Saanich Canning Company was settler-controlled and, from its opening in 1905 until its closure in 1942, was among the largest clam canaries in BC.

The harvesters supplying clams to canneries shifted from being predominantly Indigenous to a more mixed group, with increasing numbers of settlers. This growing settler involvement ultimately drove the fishery to its peak and contributed to its subsequent collapse in southern British Columbia. For example, in 1912, 100 Indigenous harvesters regularly brought clams from up to 20 miles away to the Saanich cannery, but by 1938, 300 diggers were working for the Saanich cannery, with an equal number of Indigenous and settler harvesters [70,86]. Demand for clams from American canneries also brought an influx of harvesters from Washington State in the 1930s. In 1938, so many Americans were crossing the border that they were ‘making it almost impossible’ for locals in the Gulf Islands to make additional earnings from the beaches [87], although large-scale harvesting by Canadian settlers continued in this period [88].

#### (iv) Shells

Indigenous communities reuse many clam shells for myriad purposes, including returning them to beaches to improve the sediment structure and increase productivity [8,68,89]. Studies have shown that leaving shells on the beach helps bivalve populations thrive, as shell fragments create surfaces for young clams to settle on. Removing shells breaks this natural cycle [13]. In other cases, shells were used in construction, to reclaim land on the coast, create mounds or make paths more visible in the dark [47]. Clam shell deposits also add calcium and other minerals to the soil, enhancing its fertility and moisture retention, which in turn supports the growth of adjacent forests [90]. Shell deposits also hold deep cultural importance as integral components of archaeological sites, serving as the most ubiquitous material record of deep-time Indigenous practices in the region.

In the commercial clam industry, empty shells were not returned to beaches but were commodified and sold as a byproduct of the canning process (figure 4D). Shells were ground down and used in chicken feed, for road building and as grit for making stucco [70,86]. In other instances, settlers ‘mined’ cultural shell deposits near Indigenous communities. For example, in 1931, a settler sought to lease Shingle Point on Valdes Island to extract clam shells for use in cement. Shingle Point is reserve land of the Lyackson First Nation and is an example of land reclaimed using shell deposits, which also created a sheltered area for clams to grow [91]. The Lyackson First Nation firmly refused to lease the land or sell the shell deposits.

In another case from 1936, the Indian Commissioner reported that a Vancouver company intended to remove clam shells from the foreshore of reserve land near Fort Rupert on the north end of Vancouver Island [92]. Later in 1937, DFO received a request for information on clam shell deposits on Vancouver Island for potential use as chicken feed [93]. Although DFO had no specific data on shell deposits, they suggested possible sites near Ladysmith Harbour and Coffin Island. They also noted that in previous years, the Brackman Kerr Milling Company had been ‘mining’ clam shells from an extensive deposit on Penelakut Island, opposite Chemainus, which was likely a cultural shell deposit [94].

Similarly, a very large and significant ancestral K'ómoks First Nation village and cemetery site near Seal Island was mined by settlers from the 1920s to the 1950s [95]. The mining of shell deposits from Indigenous village and cemetery sites by settlers not only disrupts ecological systems, but also represents a form of structural violence, destroying and/or erasing the deep-time material records of Indigenous cultural heritage (L. Tarle 2025, personal communication)[96]. K'ómoks First Nation archaeology staff also note that ancestral village and cemetery sites contain human burials which may have been disturbed and ground up along with the shells (J. Morin, 2025, personal communication).

### (b) Ecological change

This disruption of ancestral clam garden tending led to rapid ecological degradation and overexploitation of native clam species in British Columbia, as evidenced by serial exploitation, species shifts, rapid declines in relative abundance and failure to recover after closures. In contrast, the sites for which data are available suggest that areas with ongoing Indigenous tending remained relatively stable and productive, although it is likely that many other similarly managed sites followed this pattern but lack surviving records.

#### (i) Spatial and species shifts

We found significant spatial shifts in harvesting over time, with northward shifts as clam populations in southern BC were depleted (figure 3). Initially, the largest clam landings came from the Lower Mainland (figure 3, dark purple) in southern BC, supplying markets in Vancouver [70]. Landings in this region peaked from 1915 to 1919 and declined after this. From 1905 onwards, the east coast of Southern VI (figure 3, blue) became the most productive, coinciding with the opening of several canneries in the region. Most clams came from a smaller portion of the coastline adjacent to the Saanich Cannery, which opened in 1905 (figure 2, location 3). From 1905 until 1938, Southern VI accounted for half of all clam landings in BC and in 1934, 95% of landings came from this region. Landings peaked here in 1938 and declined as clam populations were depleted.

In response to declines in the south, the industry shifted northwards. Catches in the Broughton Archipelago (figure 3, orange) increased rapidly from 1936 and remained remarkably stable from the 1930s until the late 1960s. In the 1940s, landings continued to shift northward, with catches increasing in the Central Coast (figure 3, red) and North Coast (figure 3, teal) mainland areas, both far from urban centres. Catches in these two regions peaked in 1947 and declined thereafter. This northward shift may also have been driven by beach closures in southern BC caused by HABs. Elevated toxin levels led to a harvesting ban on the west coast of Vancouver Island from 1942 to 1953, while harvesting on the east coast of Vancouver Island was sporadically prohibited from 1943 onwards [38,97]. Additionally, settler land development in southern BC likely contributed to the change, as both urban and agricultural expansion increased pollution and restricted access to the coast. Few practical or economic reasons existed for harvesters to shift north, given the distance from major canneries, urban centres and markets, strongly suggesting that the depletion and closure of southern beaches forced this shift. In 1963, all harvesting was prohibited north of the Broughton Archipelago and along much of the west coast of Vancouver Island owing to high toxin levels [39]. Following this, landings did not shift back to southern BC, indicating that most clam populations in the south remained unviable for commercial exploitation.

Additionally, we observed a transition from harvesting native to non-native clam species. Before the 1970s, butter clams were the most harvested and canned species, accounting for 90% of all commercial clam landings in most years. Littleneck clams were the second most harvested and were typically consumed fresh. A smaller number of razor clams were also harvested, with almost all landings originating from Masset beach on the north coast of Haida Gwaii (figure 2, location 6). By 1977, the introduced Manila clam had replaced native clams as the most harvested species (see electronic supplementary material for a breakdown of species landings).

#### (ii) Declines in relative abundance

We found strong evidence of declines in relative abundance from DFO-collected data on catch per unit effort. In response to complaints of clam depletion in the mid-1930s, DFO collected data on the catch per tide (CPT), or the weight of clams collected by a single digger in one tide, from 1939 onwards. The CPT figures discussed here and shown in figure 5 represent averages from multiple diggers. These CPT data represent a constant fraction of a beach’s clam population, making it a valuable indicator of clam abundance [30].

The reliability of these data varied, but the most reliable and site-specific data demonstrated decline. One consistently collected set of CPT data for butter clams on Seal Island showed dramatic declines (figure 5A). Seal Island was closed to all harvesting for 4 years before 1942 for conservation purposes and when harvesting resumed, CPT was 272.35 kg. By 1950, the CPT had dropped to 70.76 kg, a decline of 74% [24]. CPT then rebounded somewhat to 126 kg by 1962 (we have no CPT for the beach after this date). DFO analysis has shown that almost all landings from 1942 to 1950 were from two strong year-classes in 1934−1935 before the beach was closed [72]. Clam density estimates from Seal Island show a similar trend (figure 3D), with declines from an estimated 58.39 clams m^−2^ in 1940 to only 9.34 clams m^−2^ in 1961, a decline of 85%. Clam density rebounded to 22.07 clams m^−^² by 1980 but declined thereafter, with the most recent survey in 2022 recording only 17.53 clams m^−^² (A. Dalton 2024, personal communication) [51,52].

Similar declines in CPT are demonstrated for other beaches. Data for razor clams on Masset beach in Haida Gwaii shows a steep decline of 54% from 1942 to 1947 (figure 5B). Commercial harvesting only restarted at Masset in 1942 after a 10 year gap. Similarly, commercial harvesting for butter clams only began on the North Coast Mainland in 1941. In 1942, CPT there was recorded at 253 kg but dropped to 141 kg by 1947—a decline of 56% in only 5 years (figure 3C). In contrast, CPT in the Broughton Archipelago remained very stable. Commercial harvesting began in the Broughton Archipelago in 1936. In 1940, CPT was recorded at 89.5 kg, and by 1947, it was 90 kg [53].

#### (iii) Legacy of Indigenous tending

Firsthand accounts from ancestral Knowledge Holders, like Chief Kwaxsistalla Wathl’thla, tell us that ancestral tending techniques were still employed in the 1930s, but faced threat from colonial cultural persecution. Kwaxsistalla Wathl’thla himself only learned ancestral techniques because his family hid him from the police when they came to take him away to a residential school [67].

**Figure 6.**
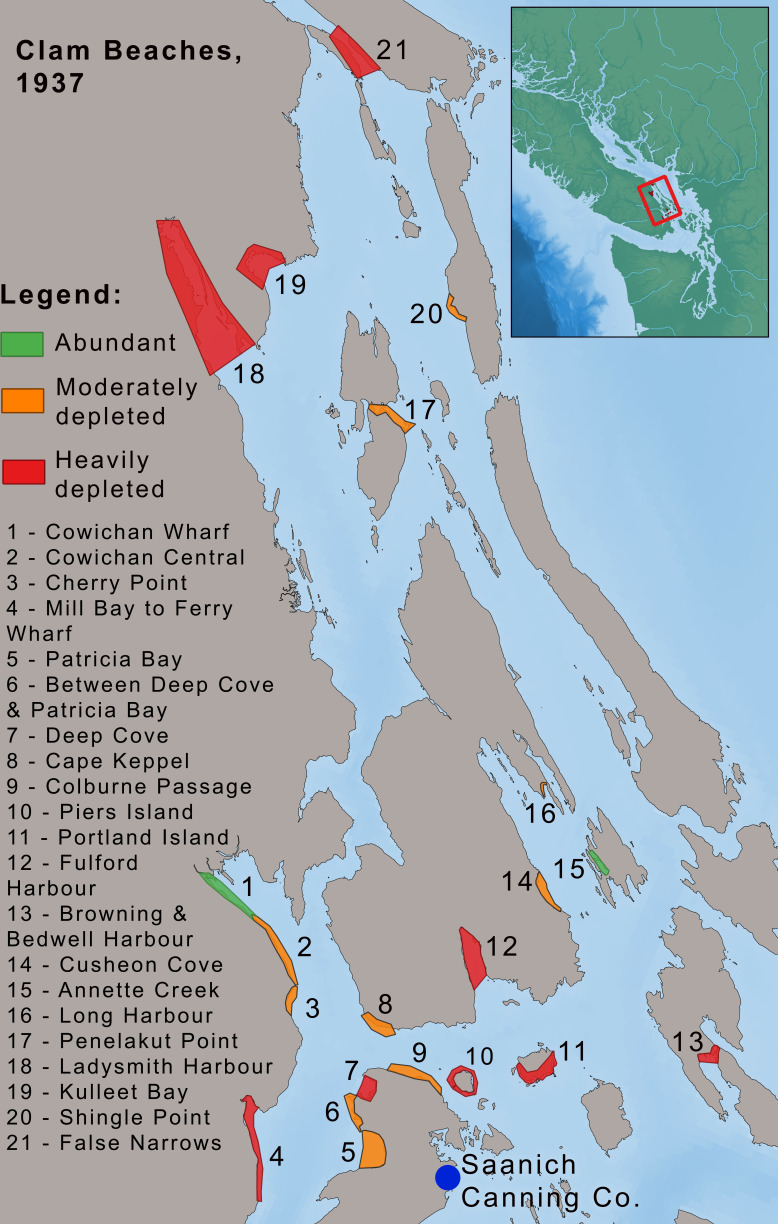
Status of beaches on the east coast of southern Vancouver Island, 1937 [98] See the supporting document (supplementary material) for more details on beaches [28].

We found additional evidence of the continued efficacy of Indigenous clam tending practices during the commercial clam fishery. DFO evidence supports the idea that clam tending prolonged the productivity of clam beaches. In 1937, DFO conducted a survey of 21 clam beaches around the Saanich Canning Company, just before landings in the area declined sharply [98] (figure 6). The survey found that almost all beaches in the area were in a depleted state. Some small beaches were still abundant with clams, but this was due to their inaccessibility (e.g. Annette Creek, location 15 in the figure). The large beach at False Narrows (location 21) was found to be ‘thoroughly dug over’ in 1937. This area had been closed to harvesting for 6 years before 1935, but when it reopened yields remained low. In contrast, the most productive beach in the survey continued to have Indigenous harvesting. The large beach at Cowichan Wharf (location 1) was located directly beside a Cowichan First Nation community who continued to tend and harvest it. DFO observed that the beach remained productive and noted depletion was ‘not as great as might be expected, or as great as local opinion would have it’ [98, p. 4]. A similar legacy of continued ancestral tending has been observed in the Broughton Archipelago, which demonstrated stable landings and CPT from the 1930s until the 1960s [78].

There is evidence that DFO scientists and other settlers were aware that the absence of clam cultivation practices could result in degradation. For example, a settler who had leased False Narrows beach found ‘that if the beds were not dug annually they would become covered by a heavy growth of barnacles and mussels, which he considered to utilise a large proportion of the available food, as well as tending to smother the clams’ [98, p. 7]. As a result, he employed local Indigenous people to harvest and tend the beach annually from 1937 onwards. On other sites, there was evidence that the loss of previous tending resulted in declines. DFO noted that the beach at the eastern end of Cowichan Bay had not been dug for some time, as evidenced by the growth of eelgrass and the presence of many dead clams, which caused ‘an obnoxious stench’ [98, p. 3]. The reports of bad smells are consistent with ancestral knowledge shared by Kwaxsistalla Wathl’thla, who said ‘When we don’t dig, the dirt it gets dark; [it] smells bad’ [67, p. 207]. The same conditions were found on other beaches, like Portland Island (location 11), suggesting these too had been tended in the past, but this had lapsed in recent times. The author of the 1937 DFO report speculated that ‘It may be that clam beds require cultivation, but his point requires investigation’ [98, p. 4].

## 5. Discussion

The shift from ancestral to settler-colonial clam fisheries in BC in the late nineteenth and early twentieth centuries devastated clam populations and severely disrupted traditional clam tending practices. This transition, driven by economic, social, legal and ecological mechanisms, led to the widespread dispossession of Indigenous clam harvesters and a paradigm shift into unsustainable commercial practices. Colonial laws and institutions systematically dismantled Indigenous governance over clam fisheries by banning cultural practices and imposing restrictions that disrupted traditional systems of resource management. These restrictions were further compounded by the imposition of a common-pool resource system, which replaced Indigenous place-based management with a framework that encouraged open access and incentivized rapid, unsustainable harvesting.

When the colonial government did lease a limited number of beaches' exclusive harvesting, Indigenous applicants were consistently denied in favour of settlers, preventing communities from maintaining exclusive rights to their ancestral clam beds. The increased prevalence of marine biotoxins has also severely disrupted clam fisheries since the 1940s, transforming a resource that traditionally nourished communities into one now dangerous for human consumption. The severity of these toxin events led to the complete closure of the northern clam fishery in 1963, a closure that remains in effect today. Persistent beach closures continue to render many clam populations unsafe, disproportionately affecting Indigenous communities [36,37]. Furthermore, the physical impacts of other industries and development on beach conditions intensified declines in clam abundance.

The loss of access to clam beds was part of a broader process of dispossession and cultural erasure that began in the late eighteenth century. European disease, colonial policies and the division of lands through the USA/Canada border in 1846 undermined the systems supporting sustainable Indigenous harvesting. Colonial policies like the Indian Act, Potlatch ban and forced residential school attendance further disrupted cultural continuity and governance. These policies and shifts in governance intensified the effects of environmental degradation, leaving many Indigenous communities unable to maintain their traditional practices and connection to the land and sea.

Our analysis reveals that the commercial exploitation of clams, fuelled by the capitalist extraction model, led to significant ecological degradation, including serial depletions and rapid declines in clam populations. Clam populations were first depleted in southern BC, prompting the industry to shift northward in search of new sources. Catch per unit effort data showed declines within a few years of commercial harvesting beginning in almost all areas, with order of magnitude declines in measured density. These values are conservative, as clam populations were already impacted when the initial data were collected. Nonetheless, they provide baseline information that can be used to guide restoration targets. These findings offer insights into the historical productivity of clam beaches and highlight the importance of securing access to traditional foods, which can enhance food security for coastal communities.

While Indigenous tending promoted long-term ecological health, DFO-regulated closures disrupted traditional practices and failed to achieve recovery. However, despite their many challenges, Indigenous communities continued to tend ancestral beaches in our study period. In the Broughton Archipelago, where Indigenous tending continued, clam abundance remained stable after the commercial industry began [78]. In southern BC, the only beach that remained abundant by DFO’s metrics was one with ongoing Indigenous tending, and those that were no longer actively tended showed distinctive signs of decline. These examples reflect the deep history of sustainable practices employed by Indigenous communities—practices rooted in thousands of years of knowledge. There are likely many other examples of beaches that continued to have Indigenous management during our period but were not uncovered in this research.

Despite the evidence that active management practised by Indigenous communities was more effective, DFO continued to apply beach closures as a conservation policy. Today, the prevailing narrative from DFO is that shifting market preferences were the primary factor behind both the decline in native clam landings and the subsequent rise of the introduced Manila clam [99]. However, the historical documents counter this narrative, exposing how it overlooks crucial factors, including the decline of Indigenous management practices and increased levels of biotoxins. Recognizing the fundamental drivers of fisheries declines is essential for making meaningful progress in restoration efforts and promoting equitable fisheries.

These findings reflect a broader pattern of species declines observed both in BC and globally when species become commercially valuable and centralized governments or agencies take over management. This process of disenfranchising the rights, cultural connections and decision-making authority away from Indigenous or local peoples has frequently caused collapses in species abundances and sharp drops in fisheries productivity [18,100–103]. Indigenous Knowledge Holders, community members, and harvesters have long recognized this pattern and have often advocated the replacement of failed Western management approaches with traditional practices and the reinstatement of their rights to manage fisheries [104,105]. While this pattern is well understood by Indigenous people in BC, the full picture of how native bivalve populations have been impacted by Western commercialization and resource management decision-making has yet to be documented from historical records. This study addresses that gap by presenting detailed historical evidence from colonial fisheries management personnel, landings records and Indigenous stewards. Historical records help illustrate how commercial overharvesting and the suppression of Indigenous-led beach tending precipitated collapse in native bivalve species.

This research underscores the role historical analysis can play in reconciliation by critically engaging with colonial archival documents. Historical records not only expose past injustices and institutional failures, such as the detrimental policies enacted by organizations like DFO, but also provide robust, detailed evidence that directly supports Indigenous narratives. Such documentation can be instrumental in contemporary contexts, guiding resource management decisions, informing restoration targets, and strengthening Indigenous rights claims [19,78].

## 6. Conclusion

By providing a more detailed understanding of the causal impacts to both habitat and native clam populations via place-based historical records, our findings provide a new detailed perspective on the long-term impacts of serial overharvesting from the settler-colonial commercial fishery as a compounding effect of the long-term dispossession of these beaches from their ancestral stewards. This information provides further support for approaches to native bivalve restoration that prioritize revitalizing Indigenous practices and presence on the beach as well as exploring innovative new techniques (such as population enhancement, sediment or shell additions) that may help to jumpstart beaches that have suffered severe declines [106–108]. While some environmental impacts may be difficult to reverse quickly, such as climate change or impacts from urban development, the increased application of Indigenous stewardship practices, Indigenous Knowledge and resource management principles is critical to the recovery of native bivalve species in British Columbia.

## Data Availability

All supplementary material is available online, including a supporting document with additional information, a spreadsheet of landings and catch per unit effort data, and a metadata list of the historical records used in this study. Supplementary material is available online [28].
